# Occurrence of pathogenic yeast **species in artisanal cheeses from Boyacá, Colombia, including fluconazole resistant isolates

**DOI:** 10.12688/f1000research.152447.3

**Published:** 2024-10-04

**Authors:** Zilpa Adriana Sánchez Quitian, Guisell Mariana Pérez Rozo, Carolina Firacative

**Affiliations:** 1 Grupo de Investigación Núcleo, Facultad de Ciencias e Ingeniería, Departamento de Biología y Microbiología, Universidad de Boyacá, Tunja, Boyacá, Colombia; 2 Programa de Bacteriología y Laboratorio Clínico, Facultad de Ciencias de la Salud, Universidad de Boyaca, Tunja, Boyacá, Colombia; 3 Studies in Translational Microbiology and Emerging Diseases (MICROS) Research Group, School of Medicine and Health Sciences, Universidad del Rosario, Bogota, Colombia

**Keywords:** Antifungal, Antimicrobial resistance, Artisanal cheeses, Pathogenic yeasts, fluconazole

## Abstract

Yeasts are widely known for their application in food production, but also because of their clinical significance. As human pathogens, several species of yeasts, mainly of the genus *Candida* and other closely related genera, are responsible for a great number of life-threatening infections. The occurrence of yeasts in cheeses, including pathogenic species, has been largely studied, yet the antifungal susceptibility of these microorganisms is rarely reported. Here, we identified the species and determined the antifungal susceptibility profile of 45 yeast *Candida* isolates recovered from artisanal cheeses from 20 cities in Boyacá, Colombia. Among the species, *Pichia fermentans * (28.9%) prevailed, followed by *Pichia kudriavzevii * (24.4%), *Kluyveromyces marxianus* (22.2%), *Clavispora lusitaniae* (11.1%), *Candida inconspicua* (6.7%) *Candida parapsilosis* (4.4%) and *Meyerozyma guillermondii* (2.2%). Notably, all seven species have been globally reported, to a greater or lesser extent, to cause fungemia and other invasive infections with high mortality rates. Remarkably, together with the intrinsically resistant *P. kudriavzevii*, most isolates of *P. fermentans, C. inconspicua* and *C. parapsilosis* were resistant to fluconazole, one of the most common drugs to treat candidiasis. Our findings highlight the importance of exploring the ecological niches of pathogenic yeasts, together with their antifungal susceptibility, considering that the emergence of resistance in non-commensal opportunistic pathogens poses a serious threat to public health.

## Introduction

The importance of yeasts is highlighted by their application in food production, as these fungi play a vital role in fermentation ( Maicas 2020). However, the clinical significance of yeasts has also been clearly established, considering that these microorganisms, as human pathogens, are able to cause life-threatening infections, particularly among older patients and those with an underlying serious condition associated with medical interventions, comorbidities or immunosuppression ( Firacative 2020). Moreover, as antifungal resistance emerges in several species of colonizing and environmental yeasts, management and treatment challenges for the infections that they cause also appear, which represents an even major problem to public health ( Fisher et al. 2022). Among the most prominent disease-causing fungi are the ascomycetous yeasts of the genus *Candida*, together with species of closely related genera formerly grouped in the “ *Candida*” clade ( Kidd et al. 2023), which are responsible for the majority of cases of invasive fungal infection in hospital settings in the world, with some species having acquired or intrinsic resistance to commonly used antifungal drugs ( Bongomin et al. 2017; Lamoth et al. 2018).

Given that several species of yeasts are frequently found not only in raw milk but also in surfaces and material related with cheese production, handling and manufacturing, the occurrence of these microorganisms in artisanal cheeses is broadly known ( Minervini et al. 2001; Mounier et al. 2006). In addition, the implications of the presence of a particular species of yeasts in these dairy products have been widely investigated, since some species can positively contribute to the characteristic taste and flavour of cheeses, while other species can spoil the product, causing off-flavours, softening, and bad odours, among others undesirable signs of spoilage ( Minervini et al. 2001; Suzzi et al. 2003; Fadda et al. 2004; Bintsis 2021). Interestingly, from the diverse assortment of yeast species that can be present in artisanal cheeses, not only species of *Candida* and closely related genera have been found, but also of the genera *Geotrichum*, *Rhodotorula*, *Saccharomyces* and *Trichosporon*, which, albeit uncommon, are increasingly causing severe disease in humans ( Bintsis 2021; Chen et al. 2021; Gil et al. 2023).

While the instances of invasive yeast infections potentially acquired from food exposure and consumption are infrequent, these may be progressively observed, given that several emerging pathogenic yeasts species are commonly recovered from various environmental sources, including food, rather than from the normal mycobiota of humans ( Cooper Jr. 2010; Benedict et al. 2016). In *Candida* bloodstream infections (BSI), particularly, the gut has been suggested as the main source of endogenous acquisition, and although atypical *Candida* species are not usually part of the gastrointestinal mycobiota, these yeasts could be transient members of the gut, acquired during feeding ( Nucci Marcio and Anaissie 2001; Auchtung et al. 2018).

As the number of patients at-risk for fungal infections increases, there is a concurrent dramatic upsurge in the number of known opportunistic fungal species that are able to cause disease. Therefore, studies like ours addressed to explore part of the wide diversity of ecological niches of human pathogenic yeasts are helpful to provide further insights into the distribution and expansion of these microorganisms. Moreover, to our knowledge, this is the first study establishing not only the occurrence but also the antifungal susceptibility of clinically relevant *Candida* species and closely related species recovered from artisanal cheeses, with the identification of resistant isolates, mainly to fluconazole. Our study contributes to the little information regarding the epidemiology, ecology and antifungal susceptibility profiles of *Pichia fermentans* (formerly *Candida lambica*), *Pichia kudriavzevii* (formerly *Candida krusei*), *Kluyveromyces marxianus* (formerly *Candida kefyr*), *Clavispora lusitaniae* (formerly *Candida lusitaniae*), *Candida inconspicua, Candida parapsilosis* and *Meyerozyma guilliermondii* (formerly *Candida guilliermondii*) ( Kidd et al. 2023). Of notice, *C. parapsilosis* and *P. kudriavzevii* are in the high and medium priority group, respectively, of the recently issued World Health Organization (WHO) fungal priority pathogens list ( WHO 2022).

## Methods

### Isolates

**Figure 1.**
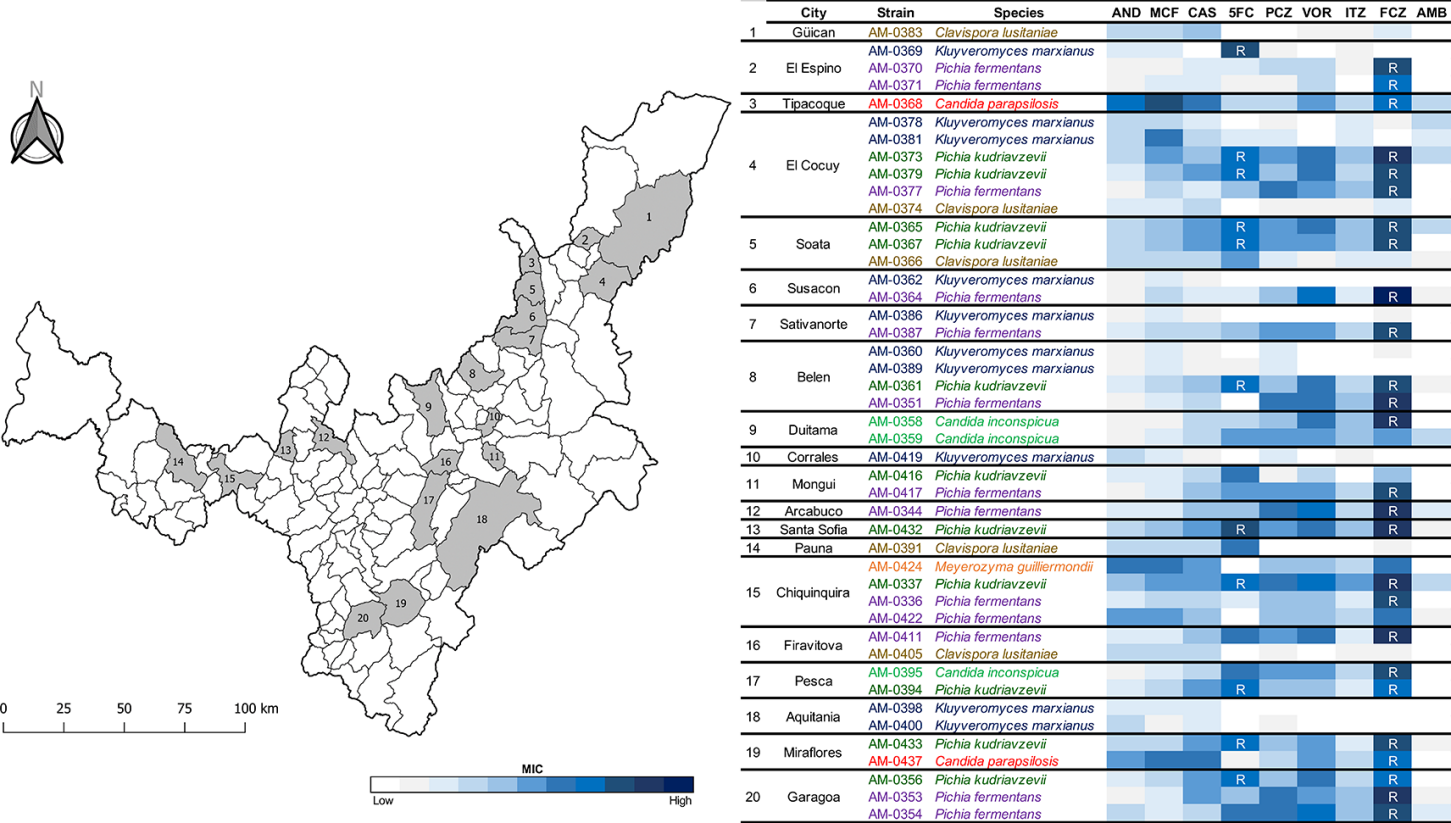
Distribution of pathogenic yeast species recovered from artisanal cheeses in Boyacá, Colombia.

Forty-five isolates of yeast species previously recovered from 29 artisanal cheeses in 20 cities in Boyacá, Colombia ( Figure 1) and belonging to the Collection of Fungi and Microorganisms of Universidad de Boyacá (UBCHM), were studied. A unique isolate of a yeast species was recovered from 15 cheeses, while two different isolates were recovered from 12 cheeses and three different isolates from two cheeses. Isolates were identified by matrix-assisted laser desorption/ionization time-off-flight (MALDI-TOF) mass spectrometry using the MALDI Biotyper ^®^ (Bruker Daltonics Inc., Germany).

Isolates were kept at -80 °C in 2 ml of 10% ultra-pure glycerol (Thermo Fisher Scientific, catalogue number 15514011) and were cultured in Sabouraud dextrose agar (SDA) (BD DIFCO, catalogue number 210950) at 35 °C for 24 h prior to the experiments.

### Antifungal susceptibility testing

The colorimetric broth microdilution test, Sensititre ^®^ YeastOne ^®^ (Thermo Fisher Scientific, catalogue number YO9), was used to determine the susceptibility of the isolates to anidulafungin (AND), micafungin (MCF), caspofungin (CAS), 5-fluorocytosine (5FC), posaconazole (PCZ), voriconazole (VOR), itraconazole (ITZ), fluconazole (FCZ) and amphotericin B (AMB), which are lyophilized in each plate, following the manufacturer’s instructions. In brief, each isolate was grown on SDA for 24 h at 35 °C. Subsequently, a yeast inoculum was prepared, per isolate, in 5 ml of sterile water and adjusted to the 0.5 McFarland standard (1-5 × 10 ^6^ cells/ml). From this cell suspension, 20 μl were mixed thoroughly with 11 ml of YeastOne ^®^ inoculum broth (Thermo Fisher Scientific, catalogue number Y3462) to obtain a final concentration of 1.8-9 × 10 ^3^ cells/ml. From the last suspension, 100 μl were served into each well of a Sensititre ^®^ YeastOne ^®^ plate. Plates were sealed and incubated at 35 °C for 24 h. Minimum inhibitory concentration (MIC) values were defined as the lowest concentration of each antifungal that prevented the development of a pink or fuchsia colour, this is, the first blue well (no growth) for amphotericin B, or the first purple well (growth inhibition) or blue well (no growth) for echinocandins, 5-fluorocytosine and azoles ( Espinel-Ingroff et al. 1999). The quality control strains of *Candida krusei* ATCC ^®^ 6258 and *Candida parapsilosis* ATCC ^®^ 22019 were used following the M27M44S guideline of the Clinical and Laboratory Standards Institute (CLSI) ( CLSI 2022).

Susceptible or resistant isolates to certain antifungal drug were identified, when available, with the clinical breakpoints per species of *Candida* and per drug, as established by the CLSI and other studies on the antifungal susceptibility of rare yeasts ( Borman et al. 2019; CLSI 2022). Per species of yeast with five or more isolates, and per antifungal drug, the frequency of MIC values was determined and the geometric mean MIC was calculated. Using the Mann–Whitney test, the differences in MIC values between species were established, per antifungal drug, with the software GraphPad Prism 9 ( https://www.graphpad.com/, La Jolla, CA, USA). *p*-values <0.05 were considered statistically significant.

## Results

### *P. fermentans* prevails among the species of yeasts recovered from artisanal cheeses

Among the 45 isolates included in this study, seven species of *Candida* and other closely related genera were identified. From these, *P. fermentans* was the most common species, with 13 (28.9%) isolates, followed by *P. kudriavzevii* with 11 (24.4%), *K. marxianus* with 10 (22.2%), *C. lusitaniae* with five (11.1%), *C. inconspicua* with three (6.7%), *C. parapsilosis* with two (4.4%) and *M. guilliermondii* with one (2.2%) isolate. From 10 cheeses with two isolates and the two cheeses with three isolates, two different species of yeasts were identified. The occurrence of the species did not differ depending on the city.

Even though all seven species recovered from artisanal cheeses have been reported as human pathogens, *C. parapsilosis, P. kudriavzevii* and *M. guilliermondii* are of major clinical relevance, as they are responsible for larger proportions of cases of candidemia and other forms of invasive candidiasis in different countries around the world ( Table 1). To a lesser extent, *C. lusitaniae*, *K. marxianus*, *C. inconspicua* and *P. fermentans* have been identified causing invasive fungal infection in humans.

**Table 1.** Ecological characteristic and estimated percentage causing invasive candidiasis of yeast species recovered from artisanal cheeses.

Current name	Previous name	n (%)	Ecology	Percentage
*Pichia fermentans*	*Candida lambica*	13 (28.9%)	Widely distributed in nature and often found in foods and fruit juice, as well as being associated with humans and animals.	Infrequent
*Pichia kudriavzevii* ^1^	*Candida krusei*	11 (24.4%)	Widely distributed in nature often occurring in soil, on fruits and in various natural fermentations.	2.5–2.7%
*Kluyveromyces marxianus*	*Candida kefyr*	10 (22.2%)	Mostly isolated from foods and beverages, especially dairy products, but also from decaying plant tissue and associated insects.	0.16%
*Clavispora lusitaniae*	*Candida lusitaniae*	5 (11.1%)	The ecological niche is ill-defined. Recovered from necrotic cactus tissue and reported as the most abundant species in leaves from agave for tequila production.	1.1%
*Candida inconspicua*	*None*	3 (6.7%)	A significant component of the yeast community in various cheeses.	0.049%
*Candida parapsilosis* ^2^	*None*	2 (4.4%)	Poorly understood. The species has been recovered sporadically from a variety of substrates and localities.	13–26.5%.
*Meyerozyma guilliermondii*	*Candida guilliermondii*	1 (2.2%)	Widely distributed in nature. Recovered from insect frass, flowers, fruits and other food products. Opportunistic pathogen of animals.	0.79–6.5%

The table was generated with publicly available data ( Pfaller and Diekema 2004; Kurtzman et al. 2011; Nucci M. et al. 2013; Arendrup et al. 2023).

^1^ Medium and

^2^ high priority in the World Health Organization fungal priority pathogens list ( WHO 2022).

### Resistance to fluconazole was identified in various species of yeasts recovered from artisanal cheeses

The majority of yeasts isolates from this study were susceptible to the echinocandins tested, to most azoles and to amphotericin B, according to the CLSI breakpoints and other studies ( Borman et al. 2019; CLSI 2022) ( Table 2). However, 12 isolates (92.3%) of *P. fermentans*, the most common species recovered, 10 (90.9%) of *P. kudriavzevii*, two (66.7%) of *C. inconspicua* and two (100%) of *C. parapsilosis* were resistant (R) to fluconazole (MIC ≥16 μg/ml) ( Figure 1). In addition, the 10 fluconazole resistant isolates of *P. kudriavzevii* had concomitantly decreased susceptibility to 5-fluorocytosine (MIC ≥8 μg/ml). Intermediate susceptibility to 5-fluorocytosine (MIC = 16 μg/ml) was as well identified in one (10%) isolate of *K. marxianus* ( Pfaller et al. 2002). Notably, fluconazole resistant isolates were identified in 16 (80%) of the 20 studied cities in Boyacá.

**Table 2.** Distribution, per species and antifungal, of the minimum inhibitory concentration (MIC) values of yeasts isolates.

				No. of isolates at MIC value (μg/ml) ^1^														
Antifungal ^2^	Species	n	GM ^3^	0.0078	0.0156	0.0313	0.0625	0.125	0.25	0.5	1	2	4	8	16	32	64	128
AND	*P. fermentans*	13	0.02663		7	4	1		1									
	*P. kudriavzevii*	11	0.04858			5	5	1										
	*K. marxianus*	10	0.03125		4	2	4											
	*C. lusitaniae*	5	0.05441			1	4											
	*C. inconspicua*	3	-		3													
	*C. parapsilosis*	2	-						1		1							
	*M. guilliermondii*	1	-							1								
MCF	*P. fermentans*	13	0.04303		1	7	4		1									
	*P. kudriavzevii*	11	0.09715			1	4	4	2									
	*K. marxianus*	10	0.04123		1	7	1			1								
	*C. lusitaniae*	5	0.05441			1	4											
	*C. inconspicua*	3	-		1	2												
	*C. parapsilosis*	2	-							1		1						
	*M. guilliermondii*	1	-							1								
CAS	*P. fermentans*	13	0.06953			4	4	4	1									
	*P. kudriavzevii*	11	0.1943				1	2	8									
	*K. marxianus*	10	0.02062	1	5	3	1											
	*C. lusitaniae*	5	0.07179				4	1										
	*C. inconspicua*	3	-				3											
	*C. parapsilosis*	2	-							2								
	*M. guilliermondii*	1	-						1									
5FC	*P. fermentans*	13	0.5274				2	1	3		4	1	2					
	*P. kudriavzevii*	11	8										1	**9**	**1**			
	*K. marxianus*	10	0.134				7	1	1						**1**			
	*C. lusitaniae*	5	0.2872				3					1	1					
	*C. inconspicua*	3	-							1		1	1					
	*C. parapsilosis*	2	-					1		1								
	*M. guilliermondii*	1	-				1											
PCZ	*P. fermentans*	13	0.202		1		1	3	3	5								
	*P. kudriavzevii*	11	0.151		1			5	4	1								
	*K. marxianus*	10	0.01675	3	3	4												
	*C. lusitaniae*	5	0.0136	2	2	1												
	*C. inconspicua*	3	-					1	2									
	*C. parapsilosis*	2	-				2											
	*M. guilliermondii*	1	-					1										
VOR	*P. fermentans*	13	0.2781				2	2	4	2	3							
	*P. kudriavzevii*	11	0.3426				1	1	2	6	1							
	*K. marxianus*	10	0.007813	10														
	*C. lusitaniae*	5	0.001184	2	3													
	*C. inconspicua*	3	-						2	1								
	*C. parapsilosis*	2	-						2									
	*M. guilliermondii*	1	-					1										
ITZ	*P. fermentans*	13	0.101		1	1	3	4	4									
	*P. kudriavzevii*	11	0.1331		1		2	3	4	1								
	*K. marxianus*	10	0.02368		6	2	2											
	*C. lusitaniae*	5	0.03125		1	3	1											
	*C. inconspicua*	3	-					1	2									
	*C. parapsilosis*	2	-					2										
	*M. guilliermondii*	1	-					1										
FCZ	*P. fermentans*	13	37.55											1	**1**	**6**	**4**	**1**
	*P. kudriavzevii*	11	26.49									1			**2**	**5**	**3**	
	*K. marxianus*	10	0.1539					7	3									
	*C. lusitaniae*	5	0.3789						2	3								
	*C. inconspicua*	3	-										1			**1**	**1**	
	*C. parapsilosis*	2	-												**2**			
	*M. guilliermondii*	1	-											1				
AMB	*P. fermentans*	13	0.1816					8	3	2								
	*P. kudriavzevii*	11	0.2663					6	2		3							
	*K. marxianus*	10	0.1768					8		1	1							
	*C. lusitaniae*	5	0.1436					4	1									
	*C. inconspicua*	3	-					2				1						
	*C. parapsilosis*	2	-					1			1							
	*M. guilliermondii*	1	-					1										

^1^ The modal MIC for each distribution is underlined. Resistant isolates are in bold.

^2^ AND: anidulafungin; MCF: Micafungin; CAS: caspofungin; 5FC: 5-fluorocytosine; PCZ: Posaconazole; VOR: Voriconazole; ITZ: Itraconazole; FCZ: Fluconazole; AMB: Amphotericin-B.

^3^ GM: Geometric mean in μg/ml calculated with five or more isolates.

When comparing the geometric mean MIC among *P. fermentans*, *P. kudriavzevii*, *K. marxianus* and *C. lusitaniae*, per antifungal tested ( Table 2), it was possible to establish that *P. kudriavzevii* was less susceptible to caspofungin, micafungin and 5-fluorocytosine than *P. fermentans*, *K. marxianus* and *C. lusitaniae* ( *p* < 0.05). Additionally, both *P. fermentans* and *P. kudriavzevii* were less susceptible to anidulafungin and all azoles than *K. marxianus* and *C. lusitaniae* ( *p* < 0.05). The susceptibility of the studied isolates to amphotericin B did not differ depending on these four species of yeasts, with all studied isolates being susceptible to this polyene.

## Discussion

The identification of nonclinical reservoirs of human pathogenic yeasts is of upmost importance, since these might serve as a source of transmission and dissemination of invasive disease. In fact, the full contribution of the environmental reservoirs of *Candida* species and closely related yeasts to the well documented shifting epidemiology of candidiasis worldwide, remains largely uncharacterized ( Lamoth et al. 2018). Here, we not only describe the occurrence of pathogenic yeast species from artisanal cheeses, but we also report the identification of about 60% of the isolates with resistance to fluconazole, some of them with concomitant reduced susceptibility to 5-fluorocytosine.

In our study, *P. fermenetans* (formerly *C. lambica*) was recovered in almost a third of samples, which is not surprising as this species was firstly isolated from butter milk in The Netherlands and since, it has been commonly encountered in different dairy products such as hard and white-brined cheeses as well as in fermented food ( Kurtzman et al. 2011; Bintsis 2021). Even though there have been very few cases of human disease by *P. fermentans*, this yeast has been able to cause fungemia, which is one of the most severe manifestations of invasive candidiasis, with high mortality rates ( Pfaller and Diekema 2004; Vervaeke et al. 2008; Noni et al. 2020). Moreover, the resistance to fluconazole that characterises the isolates of this species, which agrees with our findings, could hinder treatment and lead to an inappropriate management, hence to a poor prognosis in patients with these infections ( Borman et al. 2019).

*P. kudriavzevii* (formerly *C. krusei*), which has emerged in the last years as a significant opportunistic pathogen affecting patients with hematologic malignancies and transplant recipients worldwide ( Pfaller et al. 2008; Jamiu et al. 2021), was also commonly found in artisanal cheeses, as reported previously ( Wanderley et al. 2013; Banjara et al. 2015). In Colombia, *P. kudriavzevii* is the fifth most common yeast species causing BSI, accounting for about 2.2% of all cases, as occurring worldwide ( Pfaller and Diekema 2004; Cortes et al. 2020). Importantly, *P. kudriavzevii* has intrinsic resistance to fluconazole, as found in our isolates, reduced susceptibility to 5-fluorocytosine and is rapidly developing acquired resistance to other antifungal drugs, making it a multidrug-resistant pathogen, very difficult to treat ( Pfaller et al. 2002, 2008).

Another emerging pathogen causing BSI in patients with blood cancer is *K. marxianus* (formerly *C. kefyr*) ( Reuter et al. 2005; Dufresne et al. 2014), which accounted for about 23% of isolates recovered from our artisanal cheeses. In a global study of candidemia, *K. marxianus* was the nineth most prevalent species of yeasts, causing 0.16% of all cases ( Pfaller and Diekema 2004), and its incidence was suggested to be influenced by exogenous exposure to yogurt and other milk products ( Dufresne et al. 2014). Even though the ecology of this yeast is not fully understood, *K. marxianus* has been occasionally recovered from blue-veined cheeses and other dairy foods, as well as from fruits, plant material and even plastic devices ( Kurtzman et al. 2011; Dufresne et al. 2014; Bintsis 2021). While resistance to antifungals is not common in *K. marxianus*, we report an isolate with intermediate susceptibility to 5-fluorocytosine, which emphasises the importance of monitoring the emergence of antifungal resistance in uncommon species ( Pfaller et al. 2002).

Recovered from semi-hard, white brined and other cheeses, as well as from agave leaves ( Wanderley et al. 2013; Bintsis 2021), *Clavispora lusitaniae* (formerly *Candida lusitaniae*) was the fourth most common species recovered in our study. Known for its low susceptibility to fluconazole and amphotericin B, and its ability to acquire antifungal drug resistance within days of treatment, this species has been recognized as a human pathogen for more than four decades ( Scott et al. 2023; Angiolella et al. 2024). In fact, in a global surveillance, *C. lusitaniae* accounted for about 1.1% of all the cases of candidemia, affecting mainly immunocompromised patients with underlying malignancies ( Pfaller and Diekema 2004).

Commonly recovered from lactic products, including milk, cheeses, or butter ( Minervini et al. 2001; Suzzi et al. 2003; Bintsis 2021), *C. inconspicua* is a species that has been rarely recovered from clinical samples. Accounting for less than 0.05% of all cases of candidemia globally ( Pfaller and Diekema 2004), this yeast, together with other rare yeast species, shared the traits of being the cause of invasive infections and having high MIC values to fluconazole and azole derivatives ( Perez-Hansen et al. 2019), which difficult management.

While *C. parapsilosis* has been isolated from cheeses and milk products ( Suzzi et al. 2003; Wanderley et al. 2013; Banjara et al. 2015; Fröhlich-Wyder et al. 2019), as reported herein, its relevance lies in the role of this species in healthcare. In Australia, Malaysia, and many countries of Europe and Latin America, including Colombia, *C. parapsilosis* is the second most important opportunistic pathogenic yeast, after *Candida albicans*, associated with intrahospital transmission, targeting neonates, immunosuppressed and patients with indwelling catheters ( Pfaller and Diekema 2004; Nucci et al. 2013; Pappas et al. 2018; Arendrup et al. 2023; Hernández-Pabón et al. 2024). Remarkably, fluconazole and voriconazole cross-resistance has been described in *C. parapsilosis*, therefore, affected patients should be treated ideally with an echinocandin, which many times are unavailable in resource-limited countries ( Cornely et al. 2012). Together with *P. kudriavzevii*, which was placed in the medium priority group, *C. parapsilosis* is in the high priority group of the WHO fungal priority pathogens list, highlighting the need to focus attention on the perceived public health importance of these species ( WHO 2022).

*M. guilliermondii* (formerly *C. guilliermondii*), which is most often associated with onychomycosis, is the fourth most frequent species causing invasive fungal infection in critically ill patients in Argentina, Honduras and Venezuela, fifth in Colombia ( Nucci et al. 2013) and the seventh cause of BSI by *Candida* species globally ( Pfaller and Diekema 2004). Unfortunately, resistance to amphotericin B, fluconazole and itraconazole, associated with treatment failure, and reduced susceptibility to several other classes of antifungals have been reported in *M. guilliermondii* isolates ( Pfaller et al. 2006). Although no specific clinical or environmental sources for infection have been identified, *M. guilliermondii* may be transmitted from patient to patient in the hospital, particularly among those with intravascular foreign bodies ( Pfaller et al. 2006).

The emergence of uncommon, yet resistant, pathogenic *Candida* and other yeast species could be due to the selective pressure caused for a larger use of azole derivatives, particularly fluconazole, not only as antifungal prophylaxis but also as empirical therapeutics ( Pfaller and Diekema 2004). Given its low cost and low toxicity, fluconazole remains one of the most commonly prescribed antifungal drugs against candidemia and candidiasis ( Cornely et al. 2012). Moreover, the use of azoles as fungicides in agriculture, which can contribute to the appearance of antifungal-resistance in nature, is widely documented ( CDC 2019).

The rise of species that are in addition resistant to other class of antifungals, including polyenes, flucytosine and echinocandins, makes these microorganisms multidrug-resistant, as it is the case of *P. kudriavzevii*, *C. lusitaniae*, and *M. guilliermondii*, among the yeast species reported here, which further increases the risk for human health. Therefore, early detection, including the identification of environmental sources and exogenous exposure, as well as accurate species identification, are crucial to contribute to infection control. Our study provides important data on the occurrence of pathogenic *Candida* and closely related species recovered from artisanal cheeses and on the antifungal susceptibility of these, which until now is rather limited.

## Data Availability

Underlying data is deposited in Figshare: Occurrence of pathogenic yeast species in artisanal cheeses from Boyacá, Colombia, including fluconazole resistant isolates. Doi: https://doi.org/10.6084/m9.figshare.26093389 ( Sánchez-Quitian et al. 2024). This project contains the following underlying data:
- Raw Data.xlsx (antifungal susceptibility testing (AST) data for each isolate and per species with characteristics of each isolate). -
Figure 1 (Map with the isolates). Data are available under the terms of the Creative Commons Attribution 4.0 International license (CC-BY 4.0).
