# Supplementary material for: Occurrence of pathogenic yeast  species in artisanal cheeses from Boyacá, Colombia, including fluconazole resistant isolates
Source: F1000Res. 2024 Oct 4;13:789. Originally published 2024 Jul 11. [Version 3] doi: 10.12688/f1000research.152447.3 (PMC11503810; doi:10.12688/f1000research.152447.3)
Supplement: Peer review history

# Reviewer report: Reviewer response for version 3

**DOI:** 10.5256/f1000research.172814.r329206

**Published:** 2024-10-22

**Authors:** Maria Helena Figueiredo-Carvalho, Rowena Coelho

**Affiliations:** 1 Laboratório de Micologia, Instituto Nacional de Infectologia Evandro Chagas, Fundação Oswaldo Cruz., Instituto Nacional de Infectologia Evandro Chagas (INI)/Fiocruz, Rio de Janeiro, Rio de Janeiro, Brazil

I have reviewed the manuscript submitted by Sánchez Quitian ZA, Pérez Rozo GM, and Firacative C which evaluated the occurrence of pathogenic Yeast **species in artisanal cheeses from Boyacá, Colombia, including fluconazole-resistant isolates. The manuscript was written clearly and objectively.

Is the work clearly and accurately presented and does it cite the current literature?

Partly

If applicable, is the statistical analysis and its interpretation appropriate?

I cannot comment. A qualified statistician is required.

Are all the source data underlying the results available to ensure full reproducibility?

Yes

Is the study design appropriate and is the work technically sound?

Yes

Are the conclusions drawn adequately supported by the results?

Yes

Are sufficient details of methods and analysis provided to allow replication by others?

Yes

Reviewer Expertise:

Medical mycology

We confirm that we have read this submission and believe that we have an appropriate level of expertise to confirm that it is of an acceptable scientific standard.

# Reviewer report: Reviewer response for version 2

**DOI:** 10.5256/f1000research.172175.r327176

**Published:** 2024-09-28

**Authors:** Maria Helena Figueiredo-Carvalho, Rowena Coelho

**Affiliations:** 1 Laboratório de Micologia, Instituto Nacional de Infectologia Evandro Chagas, Fundação Oswaldo Cruz., Instituto Nacional de Infectologia Evandro Chagas (INI)/Fiocruz, Rio de Janeiro, Rio de Janeiro, Brazil

It's still not adequate. The suggested modifications need to be made throughout the entire text, from the abstract and keywords to the discussion.

Is the work clearly and accurately presented and does it cite the current literature?

Partly

If applicable, is the statistical analysis and its interpretation appropriate?

I cannot comment. A qualified statistician is required.

Are all the source data underlying the results available to ensure full reproducibility?

Yes

Is the study design appropriate and is the work technically sound?

Yes

Are the conclusions drawn adequately supported by the results?

Yes

Are sufficient details of methods and analysis provided to allow replication by others?

Yes

Reviewer Expertise:

Medical mycology

We confirm that we have read this submission and believe that we have an appropriate level of expertise to confirm that it is of an acceptable scientific standard, however we have significant reservations, as outlined above.

# Reviewer report: Reviewer response for version 1

**DOI:** 10.5256/f1000research.167210.r316497

**Published:** 2024-09-16

**Authors:** Maria Helena Figueiredo-Carvalho, Rowena Coelho

**Affiliations:** 1 Laboratório de Micologia, Instituto Nacional de Infectologia Evandro Chagas, Fundação Oswaldo Cruz., Instituto Nacional de Infectologia Evandro Chagas (INI)/Fiocruz, Rio de Janeiro, Rio de Janeiro, Brazil

I have reviewed the manuscript submitted by Sánchez Quitian ZA, Pérez Rozo GM, and Firacative C which evaluated the occurrence of pathogenic *Candida *species in artisanal cheeses from Boyacá, Colombia, including fluconazole-resistant isolates. The manuscript was written clearly and objectively. However, some modifications are necessary.

**Title **Occurrence of pathogenic *Candida *species in artisanal cheeses from Boyacá, Colombia, including fluconazole-resistant isolates. Suggest Yeast

**Abstract**

 …Remarkably, most isolates of *C. lambica* *C. krusei*, *C. inconspicua* and *C. parapsilosis* were resistant to fluconazole, one of the most common drugs to treat candidiasis. Suggest Remove the species *C. krusei*. This species is intrinsically resistant to fluconazole.

 Review all yeast species nomenclature. Kidd SE., 2023 [Ref-1] or MycoBank

 Current name *Pichia fermentan* *s*

 Previous name Candida lambica

*Pichia kudriavzevii *( *Candida krusei*)….

**Keywords** antifungal, antimicrobial resistance, artisanal cheeses, *Candida,* fluconazole. Suggest Yeast species or *Candida, Pichia, Meyrozyma, Clavispora, Kluyveromyces*

**Introduction:** … Our study contributes to the little information regarding the epidemiology, ecology and antifungal susceptibility profiles of *Candida lambica, Candida krusei, Candida kefyr,* *Candida lusitaniae, Candida inconspicua, Candida parapsilosis* and *Candida guillermondii*, two of which are part of the recently issued World Health Organization (WHO) fungal priority pathogens list (WHO 2022). Suggest *Candida parapsilosis* and *Pichia kudrizvzeveii* ( *Candida krusei*) hight and media priority group, respectively.

**Methods**

 Isolates

 Figure 1. Distribution of pathogenic *Candida* species recovered from artisanal cheeses in Boyacá, Colombia.

 Suggest Distribution of pathogenic Yeast species recovered from artisanal cheeses in Boyacá, Colombia.

 Antifungal susceptibility testing

 Was the antifungal test conducted in duplicate? Resistant isolates were found.

*Pichia kudrizvzeveii* ( *Candida krusei*) is intrinsically resistant to fluconazole. However, in your study, one isolate was sensitive.

 Results

  …Among the 45 isolates included in this study, seven species of Candida were identified. From these, *C. lambica (Pichia fermentans)* was the most common species, with 13 (28.9%) isolates, followed by *C. krusei* ( *Pichia kudriavzevii*) with 11 (24.4%), *C. kefyr* ( *Kluyveromyces marxianus*) with 10 (22.2%), *C. lusitaniae (Clavispora lusitaniae)* with five (11.1%), *C. inconspicua* with three (6.7%), *C. parapsilosis* with two (4.4%) and *C. guillermondii (Meyerozyma guilliermondii) *with one (2.2%)… Suggest current name (previous name)

- …To a lesser extent, *C. lusitaniae, C. kefyr, C. inconspicua and C. lambica* have been identified causing disease in humans. Suggest To a lesser extent, *C. lusitaniae, C. kefyr, C. inconspicua* *and C. lambica* have been identified causing invasive fungal infection in humans

Table 2

 AND: anidulafungin; MF: Micafungin; CAS: caspofungin; FC: 5-fluorocytosine; PZ: Posaconazole; VOR: Voriconazole; IZ: Itraconazole; FZ: Fluconazole; AB: Amphotericin-B. Suggest MCF: Micafungin; 5FC: 5-fluorocytosine; PCZ: Posaconazole; ITZ: Itraconazole; FCZ: Fluconazole; AMB: amphotericin B

 Discussion

 Update the references. In the discussion, the cited references are very outdated. I suggest using more up-to-date references on the species identified in this study.

Is the work clearly and accurately presented and does it cite the current literature?

Partly

If applicable, is the statistical analysis and its interpretation appropriate?

I cannot comment. A qualified statistician is required.

Are all the source data underlying the results available to ensure full reproducibility?

Yes

Is the study design appropriate and is the work technically sound?

Yes

Are the conclusions drawn adequately supported by the results?

Yes

Are sufficient details of methods and analysis provided to allow replication by others?

Yes

Reviewer Expertise:

Medical mycology

We confirm that we have read this submission and believe that we have an appropriate level of expertise to confirm that it is of an acceptable scientific standard, however we have significant reservations, as outlined above.

## References

[1] : Fungal Nomenclature: Managing Change is the Name of the Game. *Open Forum Infect Dis* .2023;10(1) : 10.1093/ofid/ofac559 ofac559 10.1093/ofid/ofac559 36632423 PMC9825814

# Response

**Published:** 2024-09-19

**Authors:** Carolina Firacative

**Affiliations:** Universidad del Rosario, Bogotá, Bogota, Colombia

**Reviewer 2.**

 I have reviewed the manuscript submitted by Sánchez Quitian ZA, Pérez Rozo GM, and Firacative C which evaluated the occurrence of pathogenic *Candida *species in artisanal cheeses from Boyacá, Colombia, including fluconazole-resistant isolates. The manuscript was written clearly and objectively. However, some modifications are necessary.

Answer: thanks to the reviewer for the time to read and evaluate the manuscript and for each of the valuable comments and suggestions. In the revised manuscript and along with this point-by-point response, we aimed to address all issues mentioned by the reviewer.

**Title **Occurrence of pathogenic *Candida *species in artisanal cheeses from Boyacá, Colombia, including fluconazole-resistant isolates. Suggest Yeast

Answer: following the reviewer’s suggestion, the title was changed.

**Abstract**

 …Remarkably, most isolates of *C. lambica* *C. krusei*, *C. inconspicua* and *C. parapsilosis* were resistant to fluconazole, one of the most common drugs to treat candidiasis. Suggest Remove the species *C. krusei*. This species is intrinsically resistant to fluconazole.

Answer: thanks for the suggestion. We did not remove the name of the species; however, we modified the text to highlight that *P.* *kudriavzevii* is intrinsically resistant to FCZ.

 Review all yeast species nomenclature. Kidd SE., 2023 [Ref-1] or MycoBank

 Current name *Pichia fermentans*

 Previous name Candida lambica

*Pichia kudriavzevii *( *Candida krusei*)….

Answer: thanks for the suggestion. We changed the names of all species through the text, keeping the current name according to Kidd et al. 2023. This reference is now included. The previous names are also mentioned in the introduction, table 1 and discussion.

**Keywords** antifungal, antimicrobial resistance, artisanal cheeses, *Candida,* fluconazole. Suggest Yeast species or *Candida, Pichia, Meyrozyma, Clavispora, Kluyveromyces*

Answer: following the reviewer’s suggestion, a keyword was changed. Instead of “ *Candida*” we now include “Pathogenic yeasts”

**Introduction:** … Our study contributes to the little information regarding the epidemiology, ecology and antifungal susceptibility profiles of *Candida lambica, Candida krusei, Candida kefyr,* *Candida lusitaniae, Candida inconspicua, Candida parapsilosis* and *Candida guillermondii*, two of which are part of the recently issued World Health Organization (WHO) fungal priority pathogens list (WHO 2022). Suggest *Candida parapsilosis* and *Pichia kudrizvzeveii* ( *Candida krusei*) hight and media priority group, respectively.

Answer: following the reviewer’s suggestion, we mention now the priority groups of *C. parapsilosis* and *P.* *kudrizvzeveii*.

**Methods**

 Isolates

 Figure 1. Distribution of pathogenic *Candida* species recovered from artisanal cheeses in Boyacá, Colombia.

 Suggest Distribution of pathogenic Yeast species recovered from artisanal cheeses in Boyacá, Colombia.

Answer: we followed the reviewer’s suggestion and changed “ *Candida*” for “yeasts”. Also, through the text.

 Antifungal susceptibility testing

 Was the antifungal test conducted in duplicate? Resistant isolates were found.

Answer: thanks to the reviewer for this question. AST was not conducted in duplicate as we followed carefully the manufacturer’s instructions and considered that the colorimetric method is comparable to the CLSI reference method for testing the susceptibility of *Candida* species, as reported several times. In addition, quality control was ensured by testing the CLSI-recommended quality control strains *C. parapsilosis* ATCC 22019 and *C. krusei* ATCC 6258, in each batch of the YeastOne plates.

*Pichia kudrizvzeveii* ( *Candida krusei*) is intrinsically resistant to fluconazole. However, in your study, one isolate was sensitive.

Answer: thanks to the reviewer for noticing this. Even though *P. kudrizvzeveii* has been long recognized as intrinsically resistant to FCZ, we revised the definition of this term in the CLSI manual: “IR is defined as inherent or innate (not acquired) antimicrobial resistance, which is reflected in wild-type antimicrobial patterns of all OR ALMOST ALL representative of a species”. In addition, there are a few publications reporting that some isolates of *P. kudrizvzeveii* can still have a low MIC to this azole, so not always 100% of the isolates of the species are resistant. Badiee et al 2017 reported ranges of MIC values from 2 to >64 μg/ml, Hrabovsky et al. 2017, ranges from 2 to 256 μg/ml, in a review of Pfaller et al 2006, ranges from 4 to >64 μg/ml and Kaur et al 2020 reported resistance in only 40.5% of the isolates, among other publications.

 Results

  …Among the 45 isolates included in this study, seven species of Candida were identified. From these, *C. lambica (Pichia fermentans)* was the most common species, with 13 (28.9%) isolates, followed by *C. krusei* ( *Pichia kudriavzevii*) with 11 (24.4%), *C. kefyr* ( *Kluyveromyces marxianus*) with 10 (22.2%), *C. lusitaniae (Clavispora lusitaniae)* with five (11.1%), *C. inconspicua* with three (6.7%), *C. parapsilosis* with two (4.4%) and *C. guillermondii (Meyerozyma guilliermondii) *with one (2.2%)… Suggest current name ****(previous name)

Answer: thanks for the suggestion. We changed the names of all species through the text, keeping the current name according to Kidd et al. 2023. This reference is now included. The previous names are also mentioned in the introduction, table 1 and discussion.

 …To a lesser extent, *C. lusitaniae, C. kefyr, C. inconspicua and C. lambica* have been identified causing disease in humans. Suggest To a lesser extent, *C. lusitaniae, C. kefyr, C. inconspicua* *and C. lambica* have been identified causing invasive fungal infection in humans

Answer: the change was done accordingly.

 Table 2

 AND: anidulafungin; MF: Micafungin; CAS: caspofungin; FC: 5-fluorocytosine; PZ: Posaconazole; VOR: Voriconazole; IZ: Itraconazole; FZ: Fluconazole; AB: Amphotericin-B. Suggest MCF: Micafungin; 5FC: 5-fluorocytosine; PCZ: Posaconazole; ITZ: Itraconazole; FCZ: Fluconazole; AMB: amphotericin B

Answer: the changes were done accordingly in the text, table 2 and figure.

 Discussion

 Update the references. In the discussion, the cited references are very outdated. I suggest using more up-to-date references on the species identified in this study.

Answer: thanks to the reviewer for the suggestion. However, we consider that the references are adequate. We added a new reference regarding the nomenclature of *Candida*.

# Reviewer report: Reviewer response for version 1

**DOI:** 10.5256/f1000research.167210.r316495

**Published:** 2024-08-29

**Authors:** Anastasia Spiliopoulou

**Affiliations:** 1 Department of Microbiology, University of Patras, Patras, Greece; 2 University General Hospital of Patras, Patras, Greece

In this manuscript, authors have studied the presence of *Candida* isolates in artisanal cheeses produced in Boyaca, Colombia. Their findings are quite interesting as they isolated species such as *Candida parapsilosis* and *C. krusei*, that are quite commonly isolated in invasive *Candida* human infections as well as other species, such as *C. kefyr*, *C. guillermondii*, *C. lusitaniae* etc that are more uncommonly encountered. It is well known that in many cases the source of candidiasis is the intestinal and colonization of certain *Candida* spp has been associated with invasive infections in certain patient groups. Moreover, antifungal resistance has spread in livestock farming, a finding that raises great concern. All these aspects are nicely presented and discussed in this work.

Is the work clearly and accurately presented and does it cite the current literature?

Yes

If applicable, is the statistical analysis and its interpretation appropriate?

Not applicable

Are all the source data underlying the results available to ensure full reproducibility?

No source data required

Is the study design appropriate and is the work technically sound?

Yes

Are the conclusions drawn adequately supported by the results?

Yes

Are sufficient details of methods and analysis provided to allow replication by others?

Yes

Reviewer Expertise:

Medical microbiology, Mycology, Antimicrobial resistance, Host-pathogen interactions

I confirm that I have read this submission and believe that I have an appropriate level of expertise to confirm that it is of an acceptable scientific standard.

# Response

**Published:** 2024-08-29

**Authors:** Carolina Firacative

**Affiliations:** Universidad del Rosario, Bogotá, Bogota, Colombia

Thanks to the reviewer for the time to read and evaluate our manuscript and for each of the valuable comments.
